# Phosphorus Poisoning

**Published:** 1895-11

**Authors:** 


### Phosphorus Poisoning.

The *Revue internationale de medecine et de chirurgie pratiques* for August 10th contains an abstract of an article on this subject by M. Magitot, which was published in the *Revue d'hygiene* for March, 1895. This affection, says the author, is the result of a slow and progressive absorption of white phosphorus. It is observed in the workmen in factories for the manufacture of matches. The vapors diffused in the atmosphere under the form of gaseous oxides, and also of phosphorus in a crude state, penetrate the organism through different tracts, especially the respiratory tract. The quantity absorbed varies according to the nature of the occupation, also according to the arrangement of the factories and the conditions of ventilation.

The rapidity with which these vapors penetrate the organism is very great, and in a few months they give rise to symptoms which persist for a long time. This condition leaves an impress of decay on the organism and cyanotic disturbance. It gives rise to bronchial troubles, to suffocation and to coryza. It alters the osseous system and fractures are more frequent among this class of workmen than among others. It also modifies the urinary secretion. In regard to treatment, Dr. Magitot advises the use of oil of turpentine, antiseptics and alkaline solutions. He also expresses the wish that the authorities would take legal measures to suppress this affection by forbidding the use of white phosphorus in the manufacture of matches.

